# Acephalous Monster

**Published:** 1851-03

**Authors:** G. W. Garland


*Acephalous Monster.*—As there is no truly philosophical hypothesis as to the origin or cause of those spots or marks on the skin of children when born, or other preternatural perversions of conformation—whether it be the influence of the maternal imagination upon the fœtus in utero, or other causes, I thought the following case might interest you and your readers.

Was called to attend the first labor of Mrs. T., aged 20, of a highly nervous temperament, but had always enjoyed vigor and health till the fourth week of her pregnancy, when she became highly excited by very slight causes. At times, her friends informed me, she was so unreasonable in her requests as to give them fears she would become insane; but after the first two months she became healthy and tranquil, and looked forward to the period of her confinement with firmness.

I found her, four hours after the accession of labor pain, and one hour after the membranes were ruptured, with active pains, and on examination found the os dilated to twice the size of a crown-piece, with breech presentation. After waiting a while, a second examination revealed the true nature of the presentation. Both feet had passed under the pubis, with heels resting against the thighs, while the breech was well down in the pelvis. I made several strong efforts to cause the feet to go above the pubis, but failed in each attempt. As the pelvis was evidently above the ordinary size, and pains strong, I concluded to let the labor progress, which was brought to a close without any unusual trouble or suffering; but the child was a species of that class of monsters termed *acephalous*. The entire head was wanting down to the ethmoid and sphenoid bones anteriorly, and lower than the superior surface of the cerebellum posteriorly. There was a small mass of pulp which lay over the body of the sphenoid bone, from which the optic nerves appeared to take their rise. The whole surface was covered by a thin, transparent membrane, through which the parts beneath could be seen, giving it the appearance of flesh or muscle. The same appearance was upon the breech and knees. The glutei muscles on either side were wanting. The patella of each leg, and the integuments about them, were also wanting. Both knees and breech were covered by the same kind of membrane and had the same appearance as the head. One thing I should mention here; at both knee and hip joints the legs were permanently flexed, which accounted for the difficulty in the labor.

A sheet was folded about the child, so that it was impossible for the mother to have seen it; and while waiting for a pain to remove the placenta, I asked her if she had been frightened during her pregnancy, or seen anything to excite disgust. She said immediately, "I have." "Soon after I became pregnant," said she, "I went down into a basement room, and came upon a rabbit which the cat had brought in." "Well," said I, "how did it look?" Said she, "the cat had eaten off the top of its head, knees and tail, and I was so frightened I did not get over it for days;" said she had often told her husband she feared it would injure her child some way, as she could not keep it out of her mind. When shown the child, which she was anxious to see, she exclaimed, "that head and those eyes look precisely like that rabbit's!"

I have always looked upon such monstrosities as *accidental* changes, which take place during the first stages of gestation; but this case put all my philosophy into the shade. What think you? Was this an accidental case, or did the influence of the mother's imagination cause that perversion? Yours,

G. W. GARLAND.

P. S. By-the-way, when a nævus growth does not exceed the size of a hazel-nut, it may be removed or destroyed by a daily application of tinct. iodine *strong*.—*N. H. Journal of Medicine.*

